# High compliance with dietary recommendations in a cohort of meat eaters, fish eaters, vegetarians, and vegans: results from the European Prospective Investigation into Cancer and Nutrition–Oxford study^☆^^☆☆^

**DOI:** 10.1016/j.nutres.2015.12.016

**Published:** 2016-05

**Authors:** Jakub G. Sobiecki, Paul N. Appleby, Kathryn E. Bradbury, Timothy J. Key

**Affiliations:** a Cancer Epidemiology Unit, Nuffield Department of Population Health, University of Oxford, Richard Doll Building, Roosevelt Drive, Oxford OX3 7LF, UK; b Department of Pediatrics, Nutrition and Metabolic Disorders, Children's Memorial Health Institute, Al. Dzieci Polskich 20, 04-730 Warsaw, Poland

**Keywords:** Vegetarian, Vegan, Nutrients, Nutrition assessment, Risk assessment, Cross-sectional study, AHS2, Adventist Health Study 2, AOAC, Association of Official Analytical Chemists International, BMR, basal metabolic rate, EAR, Estimated Average Requirement, EI, energy intake, EPIC, European Prospective Investigation into Cancer and Nutrition, FAO, Food and Agriculture Organization of the United Nations, FFQ, food frequency questionnaire, IHD, ischemic heart disease, IOM, Institute of Medicine, NSP, nonstarch polysaccharides, PUFA, polyunsaturated fatty acids, RE, Retinol Equivalents, RAE, Retinol Activity Equivalents, SFA, saturated fatty acids, WHO, World Health Organization

## Abstract

The aim of this study was to investigate differences in dietary intakes between 30 251 participants in the European Prospective Investigation into Cancer and Nutrition–Oxford study, comprising 18 244 meat eaters, 4 531 fish eaters, 6 673 vegetarians, and 803 vegans aged 30 to 90 years who completed semiquantitative food frequency questionnaires. We hypothesized that these groups characterized by varying degrees of animal product exclusion have significantly different intakes of many nutrients, with possible implications for dietary adequacy and compliance with population dietary goals. Nutrient intakes were estimated including fortification in foods, but excluding dietary supplements. Dietary supplementation practices were also evaluated. Highly significant differences were found in estimated nutrient intakes between meat eaters and vegans, with fish eaters and vegetarians usually having intermediate values. Meat eaters had the highest energy intakes, followed by fish eaters and vegetarians, whereas vegans had the lowest intakes. Vegans had the highest intakes of polyunsaturated fatty acids, dietary fiber, vitamins C and E, folate, magnesium, iron, and copper. Meat eaters had the highest intake of saturated fatty acids, protein, vitamin B_2_, vitamin B_12_, vitamin D, zinc, and iodine. Fish eaters had the highest intakes of calcium and selenium. There were no statistically significant differences in sodium and potassium intakes between dietary groups. With the exception of sodium intake, compliance with population dietary goals was high across diet groups. The results suggested a high prevalence of inadequacy for dietary vitamin B_12_ and iodine in vegans. The diet groups under study showed striking differences in dietary intakes, with possible implications for compliance with dietary recommendations, as well as cardiometabolic diseases risk.

## 1 Introduction

According to a joint Food and Agriculture Organization of the United Nations (FAO) and World Health Organization (WHO) expert consultation from the year 2004, “households across all regions should select predominantly plant-based diets rich in a variety of vegetables and fruits, pulses or legumes, and minimally processed starchy staple foods. The evidence that such diets will prevent or delay a significant proportion of non-communicable chronic diseases is consistent” [1]. This recommendation was reflected in recent dietary guidelines. For example, the 2010 Dietary Guidelines for Americans advised to “shift food intake patterns to a more plant-based diet that emphasizes vegetables, cooked dry beans and peas, fruits, grains, nuts, and seeds” [2].

However, the FAO/WHO consultation adds: “This [diet] should not exclude small amounts of animal foods, which make an important nutritional contribution to plant-food-based diets” [1]. Establishing the optimal balance between plant and animal foods for obtaining health benefits and nutrient adequacy of diets at a population level is an important goal for public health nutrition, and assessing the adequacy of habitual dietary intakes in vegetarians can prove valuable in accomplishing this task.

Vegetarians in Western countries have a lower risk of some noncommunicable chronic diseases compared with otherwise similar nonvegetarians, which may partially stem from the differences between their dietary intakes and those of the general population. A recent meta-analysis concluded that vegetarians have a significantly lower ischemic heart disease (IHD) mortality (29%) and overall cancer incidence (18%) than do nonvegetarians [3]. Previous studies in the European Prospective Investigation into Cancer and Nutrition (EPIC)–Oxford cohort showed associations between the vegetarian dietary pattern and lower risk of IHD [4], diverticular disease [5], cataract [6], hypertension [7], kidney stones [8], and some types of cancer [9].

Although it is generally accepted that appropriately planned vegetarian diets are nutritionally adequate for individuals during all stages of the life cycle and across all physical activity levels, concerns exist about their potential inadequacy in regard to some nutrients, especially in vegans [10]. This study aims to describe dietary intakes, dietary supplementation practices, and differences in dietary patterns of meat eaters, fish eaters, vegetarians, and vegans who were participants in a large cohort study. We hypothesized that these groups characterized by varying degrees of animal product exclusion have significantly different intakes of many nutrients, with possible implications for dietary adequacy and compliance with population dietary goals. Therefore, the objectives of the present study were to estimate and compare mean daily nutrient intakes between the 4 diet groups, estimate the prevalence of inadequate intakes based on food intakes alone, and compare the mean daily nutrient intakes with recommended group-level dietary targets.

## 2 Methods and materials

### 2.1 Study population

The EPIC-Oxford cohort study recruited more than 65 000 participants 20 years or older between 1993 and 1999. The participants are a cohort of generally health-conscious British residents adhering to 4 distinct dietary patterns: meat eaters, fish eaters, vegetarians, and vegans. A detailed description of the recruitment process and socioeconomic and lifestyle characteristics has been published elsewhere [11]. This study was conducted according to the guidelines laid down in the Declaration of Helsinki, and all procedures involving human subjects were approved by a multicenter research ethics committee. Written informed consent was obtained from all participants. Briefly, the EPIC-Oxford study is part of the EPIC study that aimed to recruit more than 400 000 men and women across European countries (equating to national cohorts in the range of 35 000-50 000 participants), based on sample size calculations suggesting sufficient power to detect statistically significant relative risks of at least 1.2 for all major cancer sites at this sample size [12]. The EPIC-Oxford cohort was designed to recruit as many vegetarians as possible and a similar number of meat eaters [11]. Participants were recruited through general practices in Oxfordshire, Buckinghamshire, and Greater Manchester, and by postal methods that aimed to recruit health-conscious people throughout the United Kingdom. Participants were categorized into 1 of 4 diet groups based on their response to questions asking whether they ate any meat, fish, eggs, and dairy products. Participants were categorized as those who eat meat (“meat eaters”), those who do not eat meat but eat fish (“fish eaters”), those who do not eat meat or fish but eat dairy products and/or eggs (“vegetarians”), and those who do not eat meat, fish, eggs, or dairy products (“vegans”).

First and second follow-up questionnaires were sent to surviving participants in 2001 to 2002 and 2007 to 2008, respectively. The present cross-sectional analysis of dietary patterns is based on data obtained from the third follow-up questionnaire sent to participants in 2010, on average, 14.3 years after recruitment (range, 10.5-18.6 years). Follow-up questionnaires were obtained from 32 423 participants, and those with reliable nutrient intake data and known diet group were included in the analysis. The exclusion criteria were as follows: estimated daily energy intake (EI) less than 3.3 MJ (3348.8 kJ [800 kcal]) or more than 16.7 MJ (16 744 kJ [4000 kcal]) for men and less than 2.1 MJ (2093 kJ [500 kcal]) or more than 14.7 MJ (14 651 kJ [3500 kcal]) for women, response rate less than 80% to the relevant questions in the food frequency questionnaire (FFQ), or unknown diet group. Data on dietary supplement use were obtained from the second follow-up questionnaire. Figure shows the participant selection for the study.

### 2.2 Assessment of diet

Participants completed questionnaires on diet and lifestyle that included a 112-item semiquantitative FFQ, based on the validated 130-item baseline FFQ [13], [14]. All questionnaires used in the EPIC-Oxford study are available online [15]. To calculate the mean daily intakes of nutrients, the frequency of consumption of each food or beverage item was multiplied by a standard portion size (based on UK Ministry of Agriculture, Fisheries and Food data [16]) and the nutrient content of the food or beverage [17], [18], [19], [20], [21], [22], [23], [24], [25], [26]; for the calcium content of soy foods, values were based on the nutritional information provided by manufacturers to reflect the composition of foods available on the market at the time of data collection. Calcium content was changed for the following foods to the values per 100 g shown in parenthesis: tofu (150 mg), soy yogurt (80 mg), calcium-fortified soy milk (120 mg), and soy cheese (125 mg).

Protein intake was calculated as grams per kilogram of body weight for participants who reported their weight at follow-up and had a plausible body mass index (between 15 and 60 kg/m^2^). Body mass index was calculated using height reported at baseline and weight reported at the third follow-up [27]. Sodium intakes were calculated without taking into account the use of table salt, about which no information was available from the third follow-up questionnaire. Data on the composition of foods in regard to n-3 and n-6 fatty acids, as well as up-to-date information on their trans-fatty acids content, were not available [17], [18], [19], [20], [21], [22], [23], [24], [25], [26] and hence not reported.

In order to assess the extent of underreporting in the analytic sample, the mean ratios of estimated daily EI to basal metabolic rate (BMR) were calculated, and the proportions of participants in each sex and diet group with EI/BMR ratios below the value 1.2, suggesting physiologically implausible EI for the maintenance of body weight [28], were reported. Values of BMR were calculated using the Schofield formula [29].

### 2.3 Dietary adequacy and reference intake values

Estimates of the prevalence of inadequate intakes of essential nutrients from food sources alone were calculated using the Estimated Average Requirement (EAR) cut-point method [30]. In women, prevalence of iron inadequacy was estimated only in the ≥51-year age group, because this method is not suitable when the distribution of requirements is skewed, as is the case in premenopausal women for this mineral. The EARs were primarily derived from the UK's Dietary Reference Values [31]. In the case of nutrients for which the EAR was not set (vitamin E, selenium, and iodine), values developed by the Food and Nutrition Board of the Institute of Medicine (IOM) were used as surrogate EARs [32], [33]. Alternative values were used in addition to the EARs for nutrients for which considerable differences exist in dietary recommendations between countries, that is, folate [34] and calcium [35], or for which vegetarian-specific recommendations exist, that is, iron and zinc [33]. The EAR for folate for adults ranges from 150 to 320 μg across countries, and the lowest value is currently recommended in the United Kingdom [36]. Similarly, British recommendations for calcium intake are at the low end of the wide 525- to 1000-mg range of existing EARs [37]. Because the bioavailability of iron and zinc from vegetarian, and especially vegan diets, is lower than from omnivorous diets, the IOM recommends multiplying the reference intake values for the former by 1.8 in order to obtain the actual requirements for vegetarians and notes that zinc requirements may be up to 50% higher for vegetarians whose diets contain generous amount of whole grains and legumes [33]. These foods are a rich source of phytate that inhibits the absorption of both zinc and iron [33].

The group mean intake goals for carbohydrate, total fat, saturated fatty acids (SFAs), polyunsaturated fatty acids (PUFAs), nonstarch polysaccharides (NSPs), and sodium were taken from the UK's Dietary Reference Values [31]. The goals for sugars and fiber were taken from the UK's Scientific Advisory Committee's on Nutrition 2015 report on carbohydrates [38], and the goal for potassium group mean intake was based on the value set by WHO and FAO [39]. The recommendation for fiber was originally expressed as intake of 30 g/d of “total fiber”—as determined by the method endorsed by the Association of Official Analytical Chemists International (AOAC) [38]. However, in all EPIC-Oxford analyses to date, NSPs were used to estimate dietary fiber intake and the NSP value is missing or unknown for less than 5% of foods used in the current analysis, compared with more than 10% for the AOAC fiber. Therefore, group mean intake goal was set as 23 g NSP/d, corresponding to 30 g of AOAC fiber [38]. The goal for sugars is expressed as 5% or less of total EI from free sugars [38]. Because of the unavailability of data on the free sugar content of foods, total sugars from beverages (including fruit juice), as well as the sweets and added sugars food group, were compared with this recommendation.

### 2.4 Statistical analyses

Age, daily energy and dietary nutrient intakes, and EI/BMR ratios were expressed as means with the corresponding SDs. For the purpose of testing the significance of differences in nutrient intakes between diet groups, mean daily dietary nutrient intakes adjusted for age (using age groups 30-39, 40-49, 50-59, 60-69, 70-79, and ≥80 years) and sex were calculated. Analysis of variance was used to examine the differences in adjusted mean intakes, with F tests used to assess the heterogeneity in mean-adjusted intakes between diet groups. Multiple pairwise comparisons with Bonferroni correction were used to determine the statistical significance of differences in mean intakes between pairs of diet groups. All analyses were performed using the STATA statistical package version 14 (StataCorp, College Station, TX, USA). *P* values less than .05 were considered statistically significant.

## 3 Results

Means and SDs of daily energy and dietary nutrient intakes, and EI/BMR ratios by sex and diet group are presented in Table 1. Sizeable differences were found in nutrient intakes between meat eaters and vegans, with fish eaters and vegetarians usually having intermediate values. These patterns were similar for men and women. Such patterns were also observed for the mean EI/BMR ratios, with meat eaters having the highest and vegans having the lowest values. The prevalence of EI underreporting (defined as the proportion of participants with EI/BMR ratio <1.2) was substantially higher in men than in women across all diet groups, ranging from 32.8% in meat-eating men to 42.5% in vegan men, compared with 19.1% in meat-eating women and 33.1% in vegan women.

Table 2 shows mean daily dietary nutrient intakes by diet group, adjusted for age and sex. Comparisons of nutrient intakes between diet groups were made using the adjusted values. Mean EIs were significantly different between diet groups. Meat eaters had the highest EIs (8742 kJ), followed by fish eaters (8486 kJ), vegetarians (8367 kJ), and vegans (8127 kJ). Protein intakes, expressed both as percentage of energy and as grams per kilogram of body weight, also differed significantly between diet groups, ranging from 17.2% and 1.28 g/kg of body weight in meat eaters to 13.1% and 0.99 g/kg of body weight in vegans. Total fat intake constituted on average 30% to 31% of EI in each diet group. There were major differences in the fat composition of the different dietary patterns. The mean proportion of energy from SFA in vegans was approximately one-third lower than that in meat eaters. Conversely, the mean proportion of energy from PUFA was 45% higher in vegans compared with meat eaters. Fish eaters and vegetarians had intermediate intakes of SFA and PUFA. Mean alcohol intake was low in all diet groups, providing approximately 3.5% of energy in meat eaters and fish eaters, 3% in vegetarians, and 2% in vegans. Mean fiber intakes, estimated as NSPs, were significantly different between diet groups; they were highest in vegans at 28.9 g/d and lowest in meat eaters at 21.7 g/d, with fish eaters and vegetarians having intermediate values of 24.9 and 25.6 g/d, respectively.

Vegans had the lowest intakes of vitamins B_2_, B_12_, and D, whereas meat eaters had the highest intakes of these nutrients. Conversely, mean intakes of vitamin C, vitamin E, and folate were highest in vegans and lowest in meat eaters. For total vitamin A, expressed as Retinol Equivalents (REs), meat eaters had a significantly higher mean intake than did the other 3 groups, which had similar values. However, when total vitamin A was estimated as Retinol Activity Equivalents (RAEs), obtained using a 1:12 factor for β-carotene to retinol conversion instead of 1:6 used in RE [30] (data not shown in the table), the mean daily intake of vegans adjusted for sex and age (623 μg) was significantly lower than those of fish eaters (705 μg) and vegetarians (701 μg), which in turn were significantly lower than that of meat eaters (1042 μg).

For minerals, calcium intakes were significantly higher in fish eaters and vegetarians than in meat eaters—by approximately 3% to 4%. Vegans had approximately 25% lower calcium intakes than did meat eaters. Almost one-third of vegans' calcium intake was provided by plant-based dairy substitutes (largely by fortified varieties), whereas dairy products contributed approximately 50% of calcium in the diets of the 3 other groups (data not shown). Vegans had the highest intakes of magnesium, iron, and copper, all of which were lowest in meat eaters, but vegans had the lowest intakes of zinc and iodine, and their mean iodine intake was only 28% of the mean intake of meat eaters. Vegetarians had a significantly lower mean intake of selenium than meat eaters and fish eaters, whose diets provided the highest amounts of this micronutrient; vegans had intermediate values. There were no statistically significant differences in sodium and potassium intakes between dietary groups.

Table 3 shows mean dietary intakes in the 4 diet groups in comparison to population dietary goals. Overall, compliance with the dietary goals was high across diet groups, with the exception of sodium. Meat eaters were the only group not to meet the goal of 23 g/d of fiber and to exceed the recommended maximum of less than 10% of energy from SFA, although by only 1.2 g of fiber and 0.4% of energy from SFA, respectively. All groups exceeded the advised less than 2400 mg daily sodium intake, although table salt was not included in the calculations of sodium intake. Total sugars from beverages and the sweets and added sugars food group provided approximately 3.5% and 2.5% of EI, respectively, in all diet groups (data not shown). These sugars were almost exclusively free sugars, and they did not constitute all free sugars consumed; thus, it is likely that all diet groups exceeded the advised 5% or less of energy from free sugars.

Table 4 shows the proportions of participants in each diet/sex group, with dietary intakes below the EARs for specific nutrients. Overall, the estimated prevalence of dietary inadequacies from food intake alone was low. Vitamin E was the only nutrient for which it was estimated that there was a high (ie, >50%) prevalence of inadequate intakes in meat eaters. Vegans had a high estimated prevalence of vitamin B_12_ and iodine inadequacy, as well as calcium inadequacy when the higher EAR for calcium was used as reference. Vegetarian women had a high estimated prevalence of selenium inadequacy, and vegans of both sexes and vegetarian men had a high estimated prevalence of zinc inadequacy when the bioavailability adjusted EAR was used as reference.

Data on supplement use from the second follow-up questionnaire are presented in Table 5. Among men, regular use of dietary supplements was most common in vegans, although the differences between diet groups were modest (range, 49.8%-60.8%). Among women, the proportion reporting regular use of any dietary supplements was similar across diet groups (range, 63.3%-68.3%). Compared with the other groups, a higher proportion of vegans reported using a vitamin B_12_ supplement, multivitamins with multiminerals, and flax/linseed. Overall, 50.1% of vegans and 38.7% vegetarians used at least 1 dietary supplement that was a source of vitamin B_12_.

## 4 Discussion

### 4.1 Key findings and implications for chronic disease risk

The present cross-sectional analysis of dietary intakes in the EPIC-Oxford cohort at third follow-up found, as we hypothesized, striking differences in nutrient intakes between meat eaters and vegans, with fish eaters and vegetarians usually having intermediate values. These findings are similar to the results of the baseline analysis of differences in nutrient intakes between these dietary patterns [11]. Overall, there was high compliance with population dietary goals and the estimated prevalence of nutritional inadequacy was generally low, reflecting the health-conscious behavior of the EPIC-Oxford cohort participants.

The observed differences in nutrient intakes between diet groups may contribute to lower rates of some diseases in vegetarians. Vegetarians and vegans had higher intakes of fiber and PUFA and lower intakes of SFA than did meat eaters—a nutrient profile associated with decreased risk of IHD [40]. These dietary characteristics, along with high intake of magnesium and low or null intake of heme iron—as also observed in vegetarians and vegans in the current analysis—are in turn associated with decreased risk of type 2 diabetes [41]. Similar results in regard to these and other nutrients were obtained in a recent analysis of baseline dietary patterns in the Adventist Health Study 2 (AHS2), with higher numbers of vegetarian and vegan participants [42].

### 4.2 Compliance with population dietary goals

Meat eaters did not meet the population dietary goal of less than 10% of energy from SFA, but exceeding it only by 0.4% of energy. It should be noted though that most recent recommendations regarding SFA intake advise individuals to “aim for a dietary pattern that achieves 5% to 6% of calories from SFA” (American Heart Association and American College of Cardiology) [43] or to consume them at a level “as low as is possible within the context of a nutritionally adequate diet” (European Food Safety Authority) [44] for maximum cardiovascular benefits. Similarly, meat eaters were only 1.2 g/d short of meeting the goal for fiber intake corresponding to 23 g NSP/d. This goal was introduced in the United Kingdom in 2015 [38] and represents an increase from the previous reference value of 18 g NSP/d [31]. Practical achievability of the new population recommendation was described as a “considerable challenge” [45]; therefore, the mean intake observed in meat eaters can be viewed as relatively high for a nonvegetarian dietary pattern. The 2015 goal for free sugars intake of 5% or less of energy [38], effectively halving the previously used limit [31], is also a challenging target. Based on sugar intake from beverages and the sweets and added sugars food group alone, all groups were likely to have mean intakes above this recommendation.

For sodium, all groups exceeded the advised less than 2400-mg level, although table salt was not included in the calculations of sodium intake. However, the estimated intakes are based on food composition tables published in the early 1990s [17], [18], [19], [20], [21], [22], [23], [24], [25], [26] and there has been an ongoing, successful salt reduction program in the United Kingdom since 2003, aiming to both reduce the salt content of processed foods and educate the public on strategies for reducing salt intake [46]. Given the health-conscious profile of the EPIC-Oxford cohort, salt-free and sodium-reduced alternatives, which were not included in the FFQ, may have been commonly used by the study participants. Thus, the actual mean sodium intakes may have been substantially lower than the estimated values. However, the opposite, that is, higher true intakes and underreporting of foods high in sodium, cannot be excluded. Also, the possible underestimation of the intake of grain products in vegetarians, and particularly in vegans (discussed below), may have underestimated their salt intake associated with these foods.

### 4.3 Limitations of dietary adequacy assessment

The assessment of dietary adequacy highlighted possible deficiencies of some essential nutrients in the cohort. When interpreting the results of the estimated prevalence of dietary inadequacies, it should be kept in mind that quantitative data on dietary supplementation were not available and so dietary supplements were not included in the calculation of nutrient intakes. Hence, the actual prevalence of inadequacies may be substantially lower for some nutrients than the results suggest and they should be interpreted with caution. This precludes us from drawing firm conclusions about the actual prevalence of dietary inadequacies.

Moreover, the calculated mean EI/BMR ratios and the associated proportions of participants with EI/BMR less than 1.2 suggest that moderate to considerable underreporting of EI was present across all sex and diet groups, which, apart from underestimating the intakes of macronutrients, may have also caused some degree of underestimation in the intakes of micronutrients. Whether or not it had a significant effect on the performance of the EAR cut-point method depends on how close the median intake of accurate reporters was to the EAR value of a given nutrient [47]. The extent of energy underreporting was considerably higher in men than in women, as well as in vegetarians, and especially vegans, compared with meat eaters. By contrast, meat eaters were found to have the lowest physical activity at baseline, with vegans having the highest, and fish eaters and vegetarians having intermediate physical activity levels [48].

Given the distinct pattern of EI underreporting in diet groups, it is likely that the FFQ underestimated these intakes in vegetarians, and especially vegans, because fixed portion sizes, developed for the general population [16], were used for calculations of dietary intakes. (The differences in underreporting between sexes suggested that these portion sizes may have been inadequate for males in general.) Because of the relatively low-energy density of staple plant foods, such as whole grains and legumes, vegans and vegetarians may be more likely to consume larger portions of staple plant foods than meat eaters. This limitation of the FFQ may also partially explain the differences in fiber intake between dietary patterns in the present study compared with AHS2. The FFQ used in AHS2 allowed participants to choose different multiples of the standard portion size (ranging from 0.5 to 1.5) for most foods in addition to reporting frequency of consumption. In AHS2, mean fiber intake (standardized to 8372 kJ/d [2000 kcal/d] and adjusted for sex, race, and age) was higher by 56.3% in strict vegetarians (vegans) compared with nonvegetarians (meat eaters) and higher by 23.4% in lacto-ovo-vegetarians (vegetarians) compared with nonvegetarians [42]. In the present study, the corresponding values (adjusted for age and sex) were 33.2% and 18.7%, respectively.

Another important consideration that needs to be made is the validity of nutrient intake estimates using the FFQ in the context of dietary adequacy assessment. Food frequency questionnaires are designed primarily for ranking participants according to nutrient or food intake, with relatively less concern for systematic errors in measuring absolute intakes [49]. Given the need for accurate individual-level intake data for the EAR cut-point method to produce unbiased estimates of prevalence of nutrient inadequacies, the IOM report on dietary assessment methods notes that “the use of semi-quantitative food frequency questionnaires is seldom appropriate for assessing the adequacy of dietary intake of groups” [30]. Both the effect of FFQ on the observed distributions of nutrient intakes and systematic underestimation or overestimation of these intakes are a potential concern for the performance of the EAR cut-point method. Nevertheless, in the absence of data obtained via other appropriate methods, nutrient intakes calculated from validated FFQs can be deemed acceptable for dietary adequacy assessment—as exemplified by an analysis of projected prevalence of inadequate nutrient intakes in Europe, carried out under the auspices of the European Micronutrient Recommendation Aligned Network of Excellence [50]. However, the FFQ used in the current analysis was not directly validated, but it was based on the validated baseline FFQ that provided reasonable estimates of usual dietary intakes [13], [14]. The differences between the 2 questionnaires were minor and consisted of 12 fewer items overall and additional vegetarian-specific foods in the current FFQ, as well as a more compact layout. These differences are unlikely to substantially alter the validity of the FFQ; nonetheless, a cautious interpretation of our findings is required.

The reported dietary intakes suggested that vegans consumed small amounts of nutrients generally found only, or predominantly, in animal foods, namely, dietary cholesterol, retinol, and vitamins B_12_ and D. This is due, in part, to the generic coding of some food items, such as cake, that are coded as being made with some animal products, whereas vegans probably consume animal-free versions. It may also be due to the occasional consumption of animal products, or to reporting consumption of animal products but meaning plant-based alternatives (eg, reporting consumption of dairy ice cream but meaning soy ice cream). This has some implications for the dietary adequacy assessment of vitamins A (in regard to estimating retinol intakes) and B_12_.

### 4.4 Micronutrients of potential concern

It is of note that although the values obtained for vitamin A, expressed as either RE or RAE, preclude firm conclusions about the prevalence of inadequacy in vegans, a significant proportion of their retinol intake is likely due to food item coding artifacts. There is no universal agreement on which vitamin A unit should be used for nutritional assessment. Retinol Equivalents are currently used in the United Kingdom and by the WHO and FAO [1], [31]. Retinol Activity Equivalents were first established in 2001 by the IOM as a vitamin A functional unit to replace RE, using a 1:12 factor for bioefficacy of β-carotene to retinol conversion instead of 1:6 used in RE [33]. In 2002 the International Vitamin A Consultative Group recommended displacing RE with RAE [51]. It is beyond the scope of this article to address the issue of the most appropriate vitamin A unit for nutritional assessment. However, the choice of the unit has major implications for dietary adequacy assessment in vegetarians and vegans. The proportional contribution of carotenoids to their total vitamin A intake is high, and carotenoids are the exclusive source of vitamin A in vegans unless fortified products or dietary supplements are used.

Retinol constituted 16% and 28% of vegan men's vitamin A intake, expressed as RE and RAE, respectively. The corresponding values in women were 14% and 24%. Added fats and sauces provided 39% of vegans' retinol intake, and vegan dairy substitutes provided 9%. Outside these food groups, within which fortification is possible (primarily in the form of margarine in the former group), actual retinol intake in vegans seems unlikely. The prevalence of inadequate total vitamin A intakes estimated using RE was very low across all diet groups, with the highest prevalences of 7.8% in vegan men and 3.2% in vegan women. Even if all estimated retinol intake in vegans was artifactual, the estimated prevalence of inadequacy would remain low. However, when RAE was used as the functional unit, 37.2% of vegan men had intakes below the EAR, and depending on the degree of overestimation of retinol intake, the actual prevalence of inadequacy may have been high.

Approximately 55% of meat eaters had estimated mean intakes of vitamin E below the EAR. However, the FFQ did not include oils, which are a good source of this vitamin, as a separate item.

The results were strongly suggestive of a high prevalence of inadequate intakes of vitamin B_12_ and iodine in vegans. Vegan foods do not naturally contain vitamin B_12_; therefore, maintaining an adequate supply in the diet requires the consumption of dietary supplements and/or fortified foods. The latter may have contributed substantially to the intakes of this vitamin in vegans, but the food tables used in the present study did not take account of such fortification in plant-based dairy alternatives. The proportion of vegans who reported regular use of vitamin B_12_ as a single supplement at the second follow-up was 20.8%, with many others taking multivitamin supplements that are likely to contain vitamin B_12_. It should be noted that a once-daily dose of this nutrient at the Reference Nutrient Intake level (the level typically found in multivitamin supplements, including some of those marketed toward vegans, whereas single nutrient supplements of vitamin B_12_ often contain considerably higher levels) may not be sufficient for maintaining adequate B_12_ status [52]. The rate of absorption of vitamin B_12_ is limited by the capacity of ileal receptors for phagocytosis of vitamin B_12_–intrinsic factor complex and decreases along with increasing amounts of this vitamin in a single meal or a single supplement administration [33]. The reference values for dietary intakes are set under the assumption of daily intakes being spread over the course of the day. Existing vegan-specific recommendations advise using doses of 5 to 10 μg daily, unless at least 3 servings of fortified foods per day are consumed on a regular basis [53]. More recent evidence suggests that higher doses may be needed for some individuals [52], especially in the elderly [54]. Further empirical data are needed to elucidate appropriate vitamin B_12_ dosing in vegans.

For iodine, the FFQ did not record the use of seaweed and iodized salt—2 potentially concentrated sources of this micronutrient. Data on iodine supplementation were not available. It should be noted that salt has not been routinely iodized in the United Kingdom, nor are there any other public health strategies to increase iodine intakes at the population level. Bath and colleagues [55] found in a survey of 6 supermarket chains across the United Kingdom that, as of 2012, iodized salt was available for purchase in 21.5% of supermarkets (weighted for market share). With the exception of one chain that had 2% to 3% of the market share, iodized salt was 5.3 to 6.4 times more expensive than standard table salt and had low iodine content (11.5 μg/g). These factors limit the potential contribution of iodized salt to iodine intake.

A cross-sectional study of 26 British vegans (11 men, 15 women) has found considerable differences in estimating iodine intake from 4-day weighed dietary records and analysis of concurrently collected 4-day duplicate diets [56]. The estimated daily intakes were 42 μg for men and 1448 μg for women, whereas the values obtained via analyses of the duplicates were 137 and 216 μg, respectively. The high estimated mean intakes in women were due to seaweed consumption in 2 participants. The authors concluded that “the use of dietary records and food tables may be considered inappropriate to reasonably estimate iodine intake in groups of individuals consuming unconventional foods not listed, or inconsistently listed, in food tables,” which is of relevance to the dietary assessment method used in the present study. A previous study of urinary iodine excretion in 30 British vegans by the same authors identified vegans as a group at risk for iodine deficiency. The effect of seaweed consumption was also significant in this study; mean iodine intake in 3 participants who consumed seaweed approached the provisional maximum tolerable daily intake [57].

There was a high estimated prevalence of inadequate intakes of zinc among vegan and vegetarian men when estimated intakes were compared with bioavailability-adjusted EAR, that is, EAR multiplied by 1.5 for vegans and vegetarians. This value was based on the IOM report on zinc requirements, which notes that “the requirement for dietary zinc may be as much as 50 per cent greater for vegetarians and particularly for strict vegetarians whose major food staples are grains and legumes” [33]. It is unlikely that the 50% figure applies to all participants in vegan and vegetarian groups, and therefore, the estimates of the prevalence of inadequacy for these groups using the bioavailability corrected EAR may represent “the worst-case scenario.”

The estimated prevalence of inadequate intakes of selenium was found to be high among vegetarian women (60.4%). It was also considerable in vegetarian men (43.1%), as well as vegan women (48.9%). However, it is generally agreed that dietary measures are not reliable for estimating selenium intake [32]. Moreover, since the publication in the late 1980s and early 1990s of the food tables used in the present study [17], [18], [19], [20], [21], [22], [23], [24], [25], [26], large variations in estimated selenium were observed in the United Kingdom—declining from 60 μg/d in 1991 to 34 μg/d in 2000, with a subsequent increase to 58 μg/d in 2006 [58]. Overall, validity of the food tables used cannot be assumed for estimating selenium intake in the present study with data collection in 2010. Biomarkers or analysis of duplicate diet samples would be necessary for appropriate dietary assessment in regard to this mineral [32].

Available data from low-selenium countries outside the United Kingdom consistently show lower biomarkers of selenium status in vegetarians and vegans compared with nonvegetarians [59], [60], [61]. Based on the analysis of toenail concentrations of this mineral in the United Kingdom, Judd and colleagues [62] suggested that vegetarians and vegans may be at increased risk for selenium deficiency, which is in agreement with our results suggesting higher prevalence of inadequate intakes in these diet groups compared with meat eaters.

### 4.5 Conclusion

The present study described differences in dietary intakes between groups with varying degrees of animal food exclusion. Striking differences were found between meat eaters and vegans, with fish eaters and vegetarians usually having intermediate values. Overall, compliance with population dietary goals was high and the estimated prevalence of dietary inadequacy was low. The major limitation of the current study was the use of an FFQ for estimating absolute nutrient intakes. Moreover, the FFQ was not directly validated, but it was a modified version of the validated baseline FFQ used approximately 14 years ago. Vegetarian and especially vegan diets appeared to be most protective against cardiometabolic diseases, based on their high fiber content and favorable fatty acids composition. The study highlighted the possibility of a high prevalence of inadequate intakes of some nutrients among vegetarians and vegans (vitamin B_12_, iodine, and possibly zinc and selenium), which emphasizes the importance of using fortified foods and/or nutritional supplements, as well as appropriate food choices, to ensure adequate intakes of these nutrients are achieved.

## Figures and Tables

**Figure:**
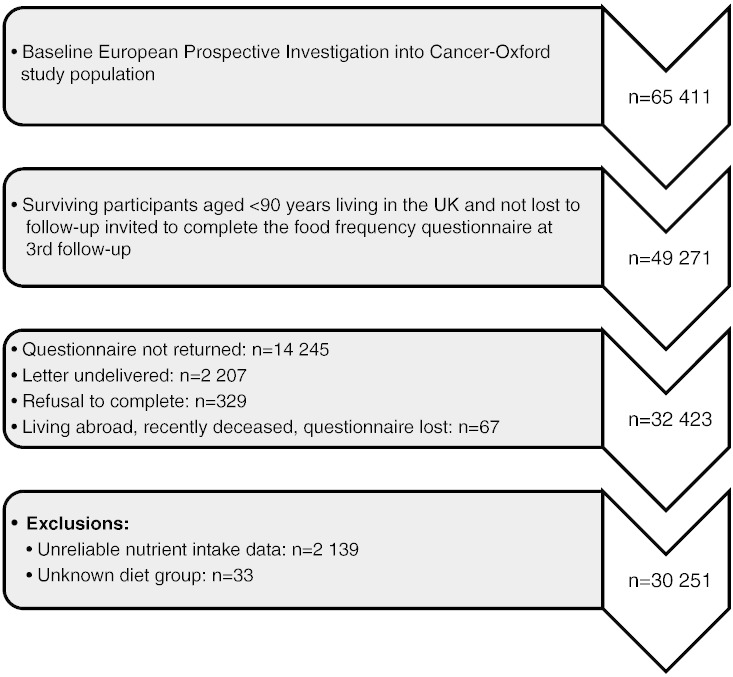
Flowchart for participant selection in the study.

**Table 1:** Age and daily dietary nutrient intakes by sex and diet group

	Meat eaters	Fish eaters	Vegetarians	Vegans
Men (n = 6365)				
n (%)	3798 (60)	782 (12)	1516 (24)	269 (4)
Age (y)	63.3 ± 11.7	58.3 ± 11.2	56.1 ± 11.0	54.2 ± 11.1
Energy (kJ)	9458 ± 2352	9249 ± 2421	9172 ± 2368	8919 ± 2650
EI/BMR ratio^a^	1.41 ± 0.41	1.36 ± 0.40	1.33 ± 0.39	1.31 ± 0.44
EI/BMR ratio <1.2 (%)^a^	32.8	40.3	40.7	42.5
% Energy from carbohydrate	48.1 ± 6.0	50.6 ± 6.0	52.3 ± 6.1	54.1 ± 7.9
% Energy from total sugars	22.9 ± 5.3	23.0 ± 5.4	22.9 ± 5.5	22.6 ± 7.2
% Energy from starch	23.3 ± 4.8	25.1 ± 5.2	26.6 ± 5.2	28.1 ± 6.9
% Energy from protein	16.5 ± 2.4	15.1 ± 2.2	13.6 ± 1.9	12.7 ± 1.9
Protein (g/kg of body weight)^a^	1.14±0.33	1.06 ± 0.31	0.95 ± 0.29	0.91 ± 0.30
% Energy from total fat	30.9 ± 4.6	30.1 ± 4.8	30.0 ± 5.3	30.4 ± 7.2
% Energy from SFA	10.4 ± 2.1	9.4 ± 2.2	9.5 ± 2.3	6.8 ± 1.8
% Energy from PUFA	6.9 ± 1.8	7.8 ± 2.1	7.9 ± 2.4	10.3 ± 3.1
P/S ratio	0.69 ± 0.23	0.87 ± 0.32	0.88 ± 0.34	1.56 ± 0.45
Cholesterol (mg)	273 ± 95	191 ± 86	154 ± 86	38 ± 30
% Energy from alcohol	4.5 ± 5.0	4.1 ± 4.7	4.1 ± 5.1	2.6 ± 3.9
Fiber (g of NSP)	22.2 ± 8.0	25.7 ± 8.3	27.0 ± 8.3	30.3 ± 9.5
Vitamin A (μg of RE)	1420 ± 678	1043 ± 438	1048 ± 416	1030 ± 515
β-Carotene equivalents (μg)	3792 ± 2020	4270 ± 2445	4292 ± 2273	5189 ± 3019
Retinol (μg)	788 ± 578	332 ± 143	332 ± 149	165 ± 111
Vitamin B_1_ (mg)	2.01 ± 0.61	2.04 ± 0.62	2.15 ± 0.70	2.42 ± 0.80
Vitamin B_2_ (mg)	2.50 ± 0.87	2.34 ± 0.81	2.35 ± 0.94	1.98 ± 1.05
Niacin (mg)	26.4 ± 7.7	22.8 ± 7.0	21.2 ± 8.0	23.8 ± 9.4
Vitamin B_6_ (mg)	2.81 ± 0.82	2.60 ± 0.78	2.52 ± 0.86	2.59 ± 0.97
Vitamin B_12_ (μg)	8.24 ± 3.10	6.59 ± 3.25	3.11 ± 2.02	0.75 ± 0.71
Folate (μg)	428 ± 143	457 ± 149	477 ± 173	539 ± 226
Vitamin C (mg)	165 ± 72	172 ± 73	171 ± 72	189 ± 85
Vitamin D (μg)	4.07 ± 1.78	3.95 ± 2.13	2.21 ± 1.33	1.96 ± 1.54
Vitamin E (mg)	12.5 ± 4.8	13.9 ± 5.2	14.2 ± 5.4	17.2 ± 7.3
Calcium (mg)	1120 ± 365	1173 ± 378	1153 ± 396	862 ± 374
Magnesium (mg)	408 ± 116	444 ± 120	451 ± 125	505 ± 157
Potassium (mg)	4302 ± 1067	4242 ± 1053	4133 ± 1008	4243 ± 1166
Sodium (mg)	2759 ± 810	2874 ± 861	2829 ± 871	2834 ± 1056
Iron (mg)	17.4 ± 5.3	17.8 ± 5.3	18.3 ± 6.0	19.9 ± 7.2
Zinc (mg)	10.9 ± 2.9	10.7 ±3.1	10.9 ± 3.2	9.4 ± 3.1
Copper (mg)	1.60 ± 0.51	1.72 ± 0.54	1.80 ± 0.57	2.23 ± 0.78
Selenium (μg)	69.3 ± 24.6	72.0 ± 27.0	54.8 ± 24.3	62.1 ± 30.4
Iodine (μg)	214.3 ± 85.6	197.4 ± (84.7)	141.0 ± 77.4	55.5 ± 40.0
Women (n = 23 886)				
n (%)	14 446 (60)	3749 (16)	5157 (22)	534 (2)
Age (y)	60.1 ± 11.8	55.7 ± 11.4	52.9 ± 11.2	51.9 ± 11.0
Energy (kJ)	8572 ± 2063	8259 ± 2048	8116 ± 2056	7862 ± 2174
EI/BMR ratio^b^	1.55 ± 0.41	1.50 ± 0.40	1.46 ± 0.39	1.42 ± 0.42
EI/BMR ratio <1.2 (%)^b^	19.1	22.7	27.2	33.1
% Energy from carbohydrate	48.0 ± 6.2	50.6 ± 6.0	52.9 ± 6.2	53.9 ± 6.8
% Energy from total sugars	23.5 ± 5.6	24.0 ± 5.7	24.6 ± 6.1	23.5 ± 7.1
% Energy from starch	22.2 ± 4.9	23.7 ± 4.9	25.1 ± 5.1	26.6 ± 6.4
% Energy from protein	17.4 ± 2.6	15.7 ± 2.3	14.0 ± 1.9	13.2 ± 1.8
Protein (g/kg of body weight)^b^	1.32 ± 0.39	1.20 ± 0.36	1.05 ± 0.33	0.99 ± 0.34
% Energy from total fat	31.4 ± 5.0	30.4 ± 5.5	29.9 ± 5.6	30.5 ± 6.2
% Energy from SFA	10.4 ± 2.2	9.4 ± 2.2	9.4 ± 2.3	6.9 ± 1.6
% Energy from PUFA	7.2 ± 2.0	7.9 ± 2.4	7.8 ± 2.4	10.3 ± 2.5
P/S ratio	0.72 ± 0.25	0.89 ± 0.34	0.88 ± 0.36	1.55 ± 0.41
Cholesterol (mg)	257 ± 92	174 ± 75	137 ± 72	32 ± 21
% Energy from alcohol	3.1 ± 3.9	3.3 ± 4.0	3.0 ± 4.2	2.3 ± 3.4
Fiber (g of NSP)	21.8 ± 7.5	24.6 ± 8.0	24.8 ± 7.8	27.7 ± 8.9
Vitamin A (μg of RE)	1408 ± 669	1093 ± 509	1054 ± 451	1048 ± 524
β-Carotene equivalents (μg)	4394 ± 2431	4797 ± 2951	4575 ± 2455	5416 ± 3125
Retinol (μg)	676 ± 529	293 ± 146	292 ± 186	145 ± 90
Vitamin B_1_ (mg)	1.88 ± 0.56	1.90 ± 0.57	1.94 ± 0.58	2.16 ± 0.65
Vitamin B_2_ (mg)	2.34 ± 0.80	2.22 ± 0.80	2.19 ± 0.82	1.69 ± 0.72
Niacin (mg)	24.8 ± 7.1	20.9 ± 6.5	18.4 ± 6.2	20.4 ± 6.7
Vitamin B_6_ (mg)	2.63 ± 0.77	2.43 ± 0.73	2.30 ± 0.72	2.31 ± 0.68
Vitamin B_12_ (μg)	7.85 ± 3.04	6.25 ± 3.17	2.96 ± 1.84	0.68 ± 0.56
Folate (μg)	413 ± 139	439 ± 154	438 ± 147	480 ± 166
Vitamin C (mg)	168 ± 73	173 ± 76	172 ± 74	187 ± 85
Vitamin D (μg)	3.78 ± 1.71	3.52 ± 2.02	1.79 ± 1.02	1.57 ± 1.05
Vitamin E (mg)	12.1 ± 4.6	13.3 ± 5.0	13.1 ± 5.1	15.6 ± 5.9
Calcium (mg)	1078 ± 341	1115 ± 360	1099 ± 368	839 ± 324
Magnesium (mg)	387 ± 108	414 ± 115	407 ± 118	452 ± 134
Potassium (mg)	4158 ± 1022	4082 ± 1056	3908 ± 1011	3972 ± 1087
Sodium (mg)	2603 ± 766	2641 ± 782	2550 ± 782	2551 ± 904
Iron (mg)	16.1 ± 4.9	16.3 ± 4.9	16.2 ± 5.0	17.6 ± 5.1
Zinc (mg)	10.4 ± 2.7	10.1 ± 2.9	10.0 ± 3.1	8.4 ± 2.8
Copper (mg)	1.54 ± 0.50	1.62 ± 0.54	1.64 ± 0.56	2.00 ± 0.66
Selenium (μg)	65.8 ± 21.8	63.6 ± 22.6	44.6 ± 18.3	51.7 ± 24.8
Iodine (μg)	213.8 ± 85.2	194.8 ± 85.9	146.1 ± 78.8	54.1 ± 40.0

Abbreviations: EI/BMR ratio, energy intake to basal metabolic rate ratio; SFA, saturated fatty acids; PUFA, polyunsaturated fatty acids; P/S ratio, polyunsaturated fat (g)/saturated fat (g); NSP, non-starch polysaccharides; RE, retinol equivalents.

Values are presented as means ± SD, or numbers and percentages.

a n = 6135: 3672 meat eaters, 749 fish eaters, 1460 vegetarians, and 254 vegans.

b n = 22 893: 13 847 meat eaters, 3596 fish eaters, 4943 vegetarians, and 507 vegans.

**Table 2:** Daily dietary nutrient intakes by diet group, adjusted by age and sex

	Meat eaters	Fish eaters	Vegetarians	Vegans
n (%)	18 244 (60)	4531 (15)	6673 (22)	803 (3)
Energy (kJ)	8742^a^	8486^b^	8367^c^	8127^d^
% Energy from carbohydrate	48.0^a^	50.7^b^	52.8^c^	54.0^d^
% Energy from total sugars	23.2^a^	24.0^b^	24.5^c^	23.7^ab^
% Energy from starch	22.5^a^	23.9^b^	25.3^c^	26.8^d^
% Energy from protein	17.2^a^	15.5^b^	14.0^c^	13.1^d^
Protein (g/kg of body weight)⁎	1.28^a^	1.17^b^	1.04^c^	0.99^d^
% Energy from total fat	31.3^a^	30.3^b^	30.0^c^	30.5^bc^
% Energy from SFA	10.4^a^	9.4^b^	9.5^b^	6.9^c^
% Energy from PUFA	7.1^a^	7.9^b^	7.8^b^	10.3^c^
P/S ratio	0.71^a^	0.88^b^	0.88^b^	1.56^c^
Cholesterol (mg)	259^a^	178^b^	143^c^	35^d^
% Energy from alcohol	3.42^a^	3.45^a^	3.16^b^	2.17^c^
Fiber (g of NSP)	21.7^a^	24.9^b^	25.6^c^	28.9^d^
Vitamin A (μg of RE)	1394^a^	1098^b^	1085^b^	1083^b^
β-Carotene equivalents (μg)	4219^a^	4725^b^	4612^b^	5524^c^
Retinol (μg)	690^a^	311^b^	316^b^	163^c^
Vitamin B_1_ (mg)	1.90^a^	1.94^b^	2.01^c^	2.26^d^
Vitamin B_2_ (mg)	2.36^a^	2.26^b^	2.25^b^	1.79^c^
Niacin (mg)	25.1^a^	21.4^b^	19.1^c^	21.5^b^
Vitamin B_6_ (mg)	2.64^a^	2.49^b^	2.38^c^	2.43^bc^
Vitamin B_12_ (μg)	7.88^a^	6.36^b^	3.09^c^	0.78^d^
Folate (μg)	413^a^	446^b^	452^b^	504^c^
Vitamin C (mg)	167^a^	174^b^	174^b^	190^c^
Vitamin D (μg)	3.79^a^	3.65^b^	1.97^c^	1.77^d^
Vitamin E (mg)	12.1^a^	13.5^b^	13.6^b^	16.3^c^
Calcium (mg)	1083^a^	1131^b^	1117^b^	848^c^
Magnesium (mg)	390^a^	421^b^	419^b^	470^c^
Potassium (mg)	4158^a^	4140^a^	4013^b^	4115^a^
Sodium (mg)	2624^a^	2701^a^	2631^a^	2645^a^
Iron (mg)	16.3^a^	16.7^b^	16.7^b^	18.3^c^
Zinc (mg)	10.5^a^	10.2^b^	10.3^b^	8.7^c^
Copper (mg)	1.55^a^	1.64^b^	1.68^c^	2.07^d^
Selenium (μg)	66.3^a^	65.5^a^	47.2^b^	54.9^c^
Iodine (μg)	212.2^a^	196.8^b^	148.1^c^	58.5^d^

Abbreviations: SFA, saturated fatty acids; PUFA, polyunsaturated fatty acids; P/S ratio, polyunsaturated fat (g)/saturated fat (g); NSP, non-starch polysaccharides; RE, retinol equivalents.

Values are presented as means adjusted for age and sex. Differences between diet groups were tested using analysis of variance. Multiple pairwise comparisons with Bonferroni correction were used to determine the statistical significance of differences in mean intakes between pairs of diet groups.

^a,b,c,d^Pairs of means in the same row that do not have a common superscript are significantly different at *P* < .05.

*P* values for heterogeneity between diet groups for all nutrients were less than .0001.

⁎ n = 29 028: 17 519 meat eaters, 4345 fish eaters, 6403 vegetarians, and 761 vegans.

**Table 3:** Nutrient intakes by diet group, compared with population dietary goals

Nutrient	Mean intakes				Goal for population mean intake^a^
	Meat eaters	Fish eaters	Vegetarians	Vegans
Carbohydrate	48.0%E	50.6%E	52.8%E	54.0%E	50%E^b^
Total fat	31.3%E	30.3%E	30.0%E	30.5%E	<33%E
SFA	10.4%E	9.4%E	9.5%E	6.8%E	<10%E
PUFA	7.1%E	7.9%E	7.8%E	10.3%E	>6%E
Fiber (NSP)	21.8 g	24.8 g	25.3 g	28.6 g	23 g/d^b^
Sodium^c^	2636 mg	2681 mg	2613 mg	2646 mg	<2400 mg/d
Potassium	4188 mg	4109 mg	3959 mg	4062 mg	>3120 mg/d^d^

Abbreviations: %E, percent of energy intake; SFA, saturated fatty acids; PUFA, polyunsaturated fatty acids; NSP, non-starch polysaccharides.

Values are presented as mean daily intakes.

a Source: unless specified otherwise, Department of Health, 1991. Dietary reference values for food, energy and nutrients in the United Kingdom.

b Source: Scientific Advisory Committee on Nutrition, 2015. Carbohydrates and health. Value converted to NSP from 30 g/d AOAC (total) fiber.

c Excluding discretionary salt use.

d Source: World Health Organization & Food and Agriculture Organization of the United Nations, 2003. Report of a Joint WHO/FAO Expert Consultation. Diet nutrition and the prevention of chronic diseases.

**Table 4:** Prevalence of inadequate intakes calculated from food sources alone, by sex and diet group

Nutrient	EAR value^a^	Meat eaters		Fish eaters		Vegetarians		Vegans	
		M	W	M	W	M	W	M	W
Protein	M, W: 0.6 g/kg of body weight	2.5%	1.2%	4.5%	1.8%	9.8%	6.0%	16.5%	8.1%
Vitamin A (RE)	M: 500 μg RE, W: 400 μg RE	3.1%	1.0%	4.5%	1.0%	4.2%	1.3%	7.8%	3.2%
As RAE	M: 500 μg RAE, W: 400 μg RAE	11.7%	5.4%	19.7%	8.3%	22.4%	9.3%	37.2%	19.9%
Vitamin E^b^	12 mg	52.8%	56.1%	39.1%	46.4%	39.1%	47.1%	23.8%	26.8%
Vitamin B_1_	0.072 mg/1000 kJ (0.3 mg/1000 kcal)	0.0%	0.0%	0.0%	0.0%	0.0%	0.0%	0.0%	0.0%
Vitamin B_2_	M: 1.0, F: 0.9 mg	1.3%	1.2%	1.7%	2.0%	2.8%	2.5%	11.5%	11.4%
Niacin	1.31 mg/1000 kJ (5.5 mg/1000 kcal)	0.1%	0.1%	0.0%	0.3%	1.1%	2.1%	0.0%	0.7%
Vitamin B_6_	13 μg/1 g of dietary protein	0.0%	0.0%	0.0%	0.0%	0.0%	0.0%	0.0%	0.0%
Vitamin B_12_	1.25 μg	0.1%	0.1%	1.2%	0.5%	17.0%	17.2%	84.8%	89.0%
Folate	150 μg	0.2%	0.4%	0.3%	0.3%	0.2%	0.3%	0.4%	0.4%
IOM EAR^c^	320 μg	21.9%	24.6%	15.9%	18.9%	14.1%	20.1%	13.8%	14.6%
Vitamin C	25 mg	0.1%	0.0%	0.0%	0.0%	0.0%	0.1%	0.0%	0.2%
Calcium	525 mg	2.5%	2.6%	1.8%	2.2%	3.0%	2.7%	17.1%	13.5%
IOM EAR^d^	M <71 y, W <51 y: 800 mg; M ≥71 y, W ≥51 y: 1000 mg	26.3%	39.4%	18.0%	33.4%	20.6%	33.4%	52.0%	64.2%
Magnesium	M: 250 mg, F: 200 mg	6.4%	1.8%	2.7%	1.0%	3.2%	1.4%	3.3%	1.1%
Iron^e^	M, W ≥51 y: 6.7 mg	0.4%	0.8%	0.5%	0.8%	0.3%	0.7%	0.7%	0.8%
Bioavailability adjusted^f^	Values multiplied by 1.8 for vegetarians and vegans	0.4%	0.8%	0.5%	0.8%	11.3%	19.2%	11.5%	12.9%
Zinc	M: 7.3 mg, W: 5.5 mg	8.3%	1.6%	9.3%	2.9%	12.3%	3.6%	26.8%	9.9%
Bioavailability adjusted^f^	Values multiplied by 1.5 for vegetarians and vegans	8.3%	1.6%	9.3%	2.9%	55.3%	29.5%	73.6%	55.8%
Selenium^b^	45 μg	12.3%	14.4%	11.9%	19.5%	43.1%	60.4%	32.7%	48.9%
Iodine^f^	95 μg	4.1%	4.0%	8.8%	8.9%	33.4%	28.7%	93.7%	92.5%

Abbreviations: EAR, Estimated Average Requirement; M, men; W, women; RE, retinol equivalents; RAE, retinol activity equivalents; IOM, Institute of Medicine.

Prevalence of inadequate intakes was estimated using the EAR cut-point method.

a Source: unless specified otherwise, Department of Health, 1991. Dietary reference values for food, energy and nutrients in the United Kingdom.

b Source: IOM, 2000. Dietary reference intakes for vitamin C, vitamin E, selenium, and carotenoids.

c Source: IOM, 1998. Dietary reference intakes for thiamin, riboflavin, niacin, Vitamin B_6_, folate, vitamin B_12_, pantothenic acid, biotin, and choline.

d Source: IOM, 1998. Dietary reference intakes for calcium and vitamin D.

e Prevalence calculated in women for the ≥51-year age group only (see Section 2.3).

f Source: IOM, 2001. Dietary reference intakes for vitamin A, vitamin K, arsenic, boron, chromium, copper, iodine, iron, manganese, molybdenum, nickel, silicon, vanadium, and zinc.

**Table 5:** Use of dietary supplements in diet and sex groups at second follow-up

Type of dietary supplement	Meat eaters	Fish eaters	Vegetarians	Vegans
Men (n = 5 103)				
n (%)	3 000 (59)	641 (13)	1 245 (24)	217 (4)
Any supplements	54.3%	51.8%	49.8%	60.8%
Any supplement containing vitamin B_12_^a^	21.2%	29.6%	33.7%	50.7%
Any multivitamin^b^	19.4%	27.6%	29.4%	35.5%
Multivitamin	11.1%	13.6%	13.5%	17.1%
Multivitamin with iron	2.9%	5.5%	6.7%	3.7%
Multivitamin with calcium	0.9%	0.5%	0.6%	2.3%
Multivitamin with multiminerals	6.9%	11.9%	12.9%	18.9%
Fish oil	34.6%	24.0%	4.4%	0.0%
Evening primrose oil	2.1%	2.8%	2.7%	1.4%
Flax/linseed	3.1%	6.7%	13.6%	26.7%
Vitamin A	2.0%	2.3%	1.6%	2.3%
Vitamin D	2.3%	1.7%	2.3%	5.5%
Vitamin E	5.5%	4.2%	3.7%	4.1%
Vitamin B_6_	3.0%	3.4%	3.4%	6.5%
Vitamin B_12_	2.8%	4.1%	6.7%	22.1%
Folic acid	3.2%	3.6%	2.5%	4.6%
Calcium	4.1%	4.8%	4.5%	8.8%
Magnesium	2.5%	4.4%	2.6%	2.8%
Iron	1.6%	2.5%	2.6%	2.6%
Zinc	6.2%	8.4%	7.3%	8.3%
Selenium	4.8%	5.6%	4.3%	7.4%
Women (n = 18 873)				
n (%)	11 238 (60)	3 020 (16)	4 164 (22)	451 (2)
Any supplements	66.8%	68.3%	63.3%	67.0%
Any supplement containing vitamin B_12_^a^	28.9%	38.3%	40.1%	49.9%
Any multivitamin^b^	25.8%	33.3%	35.1%	37.3%
Multivitamin	12.8%	12.4%	13.6%	14.4%
Multivitamin with iron	4.7%	8.5%	10.2%	4.7%
Multivitamin with calcium	1.3%	1.7%	1.0%	1.8%
Multivitamin with multiminerals	10.2%	15.3%	14.6%	20.8%
Fish oil	37.5%	26.4%	5.7%	1.3%
Evening primrose oil	13.9%	15.1%	14.2%	10.2%
Flax/linseed	37.3%	11.2%	16.7%	24.2%
Vitamin A	2.0%	2.3%	1.6%	1.1%
Vitamin D	5.1%	4.2%	3.8%	5.3%
Vitamin E	5.6%	6.2%	4.9%	3.5%
Vitamin B_6_	5.9%	8.0%	6.4%	7.8%
Vitamin B_12_	5.3%	8.2%	8.4%	20.2%
Folic acid	4.3%	4.5%	4.5%	4.4%
Calcium	14.4%	13.6%	11.2%	17.5%
Magnesium	5.9%	6.5%	5.3%	9.1%
Iron	3.4%	5.9%	6.4%	6.4%
Zinc	7.7%	10.0%	7.7%	8.9%
Selenium	5.0%	5.1%	3.8%	5.3%

Values are presented as proportions of participants reporting regular use of dietary supplements.

a Any one of the following: vitamin B_12_, multivitamin, multivitamin with iron, multivitamin with calcium, multivitamin with multiminerals.

b Any one of the following: multivitamin, multivitamin with iron, multivitamin with calcium, multivitamin with multiminerals.
